# Supplementary material for: Learning brain dynamics for decoding and predicting individual differences
Source: PLoS Comput Biol. 2021 Sep 3;17(9):e1008943. doi: 10.1371/journal.pcbi.1008943 (PMC8445454; doi:10.1371/journal.pcbi.1008943)
Supplement: S1 Fig — Euclidean distances between trajectories. In the inset, the duration of every clip is indicated in parenthesis. (PDF) [file pcbi.1008943.s001.pdf]

# S1 Fig. Euclidean distances between trajectories for all movie clips.

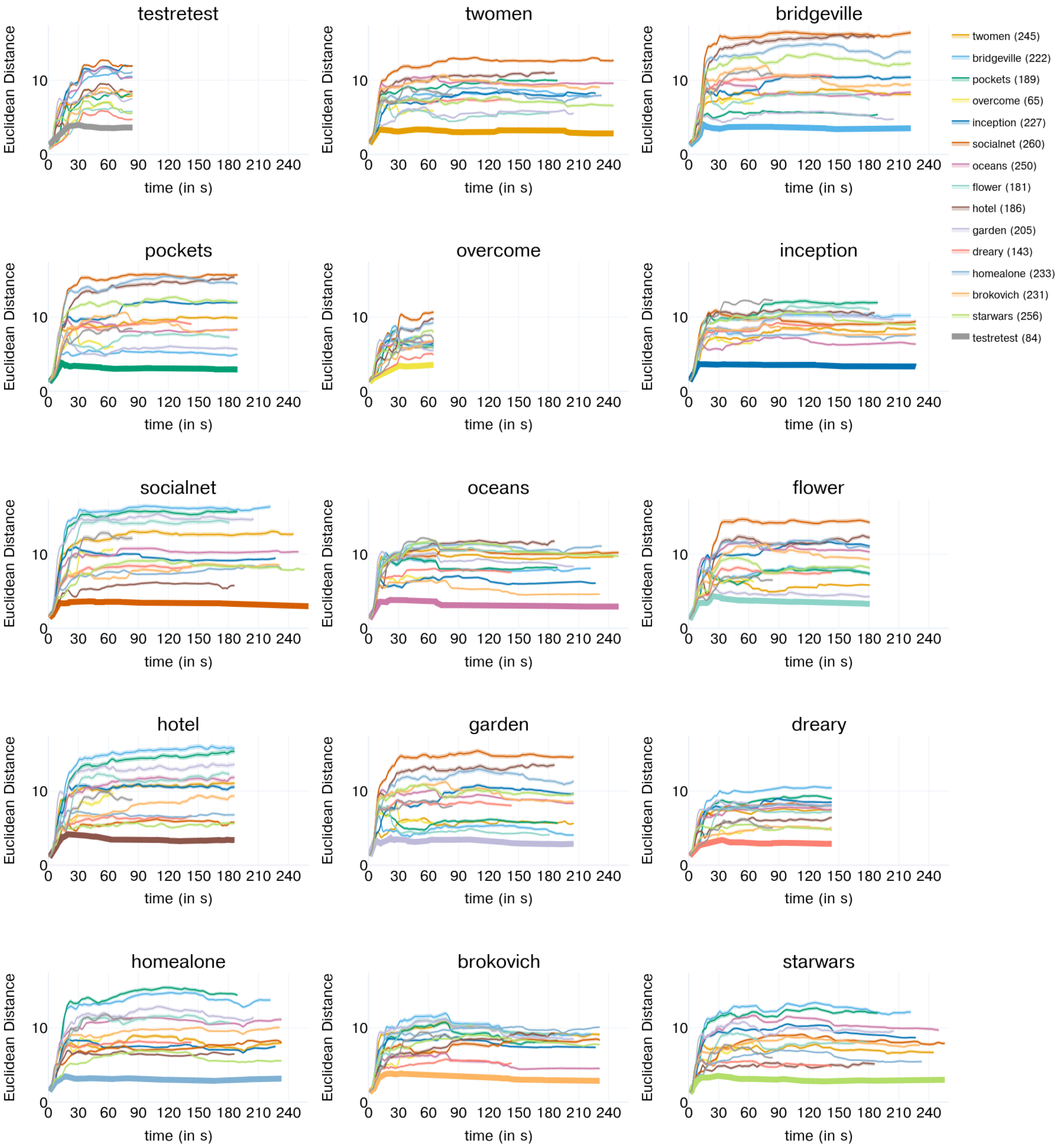


**S1 Fig.** Euclidean distances between trajectories. In the inset, the duration of every clip is indicated in parenthesis.
