# Supplementary material for: Learning brain dynamics for decoding and predicting individual differences
Source: PLoS Comput Biol. 2021 Sep 3;17(9):e1008943. doi: 10.1371/journal.pcbi.1008943 (PMC8445454; doi:10.1371/journal.pcbi.1008943)
Supplement: S2 Fig — Fluid Intelligence predictions for all movie clips. Conventions as in Fig 6 in the main text. (PDF) [file pcbi.1008943.s002.pdf]

S2 Fig. Fluid Intelligence predictions for all movie clips.

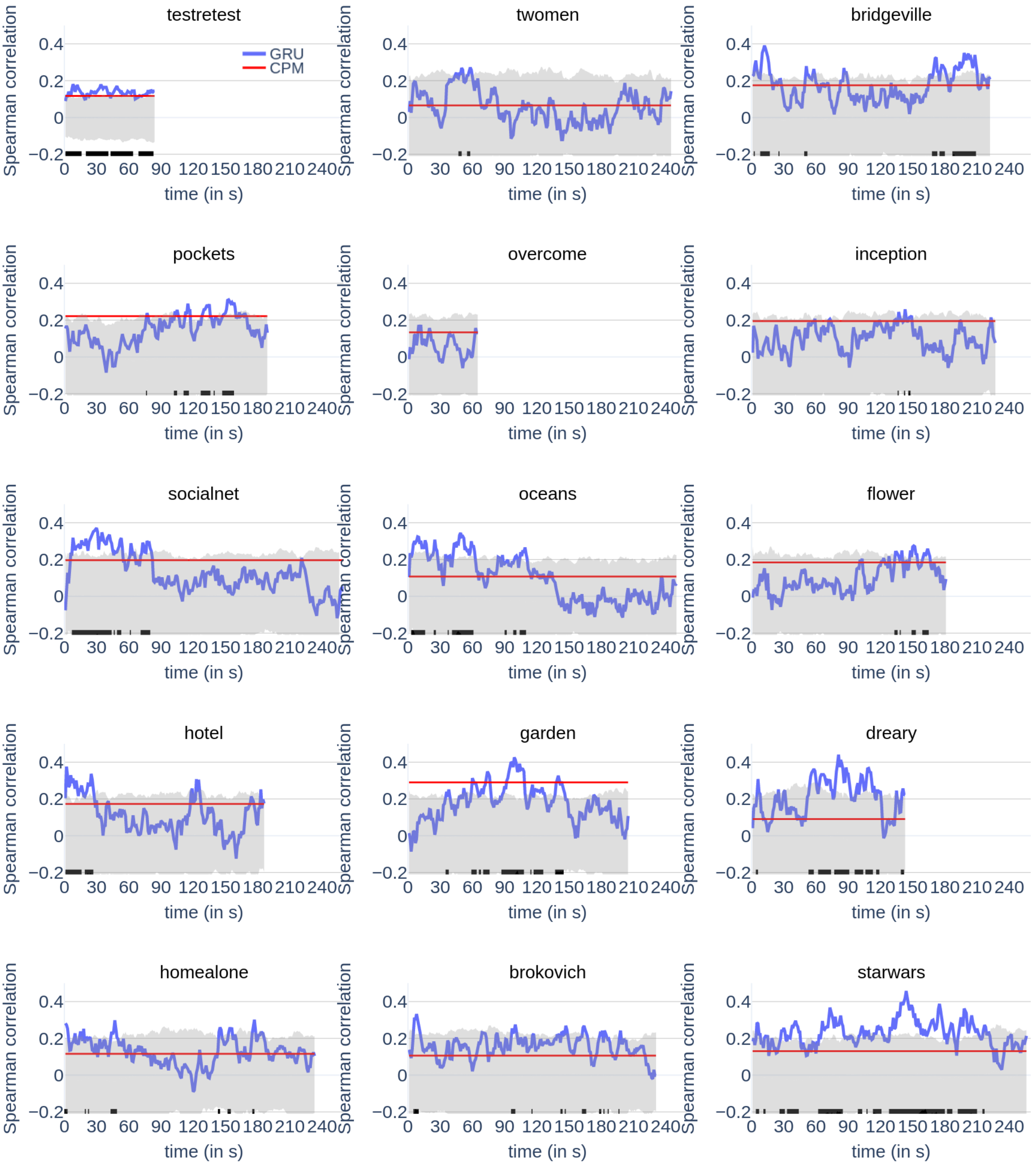

S2 Fig. Fluid Intelligence predictions for all movie clips. Conventions as in the Fig 6 in the main text.
