# Supplementary material for: Learning brain dynamics for decoding and predicting individual differences
Source: PLoS Comput Biol. 2021 Sep 3;17(9):e1008943. doi: 10.1371/journal.pcbi.1008943 (PMC8445454; doi:10.1371/journal.pcbi.1008943)
Supplement: S3 Fig — Null distributions of fluid intelligence predictions. Vertical blue lines indicate the prediction based on actual data. (PDF) [file pcbi.1008943.s003.pdf]

S3 Fig. Fluid Intelligence predictions: null distributions.

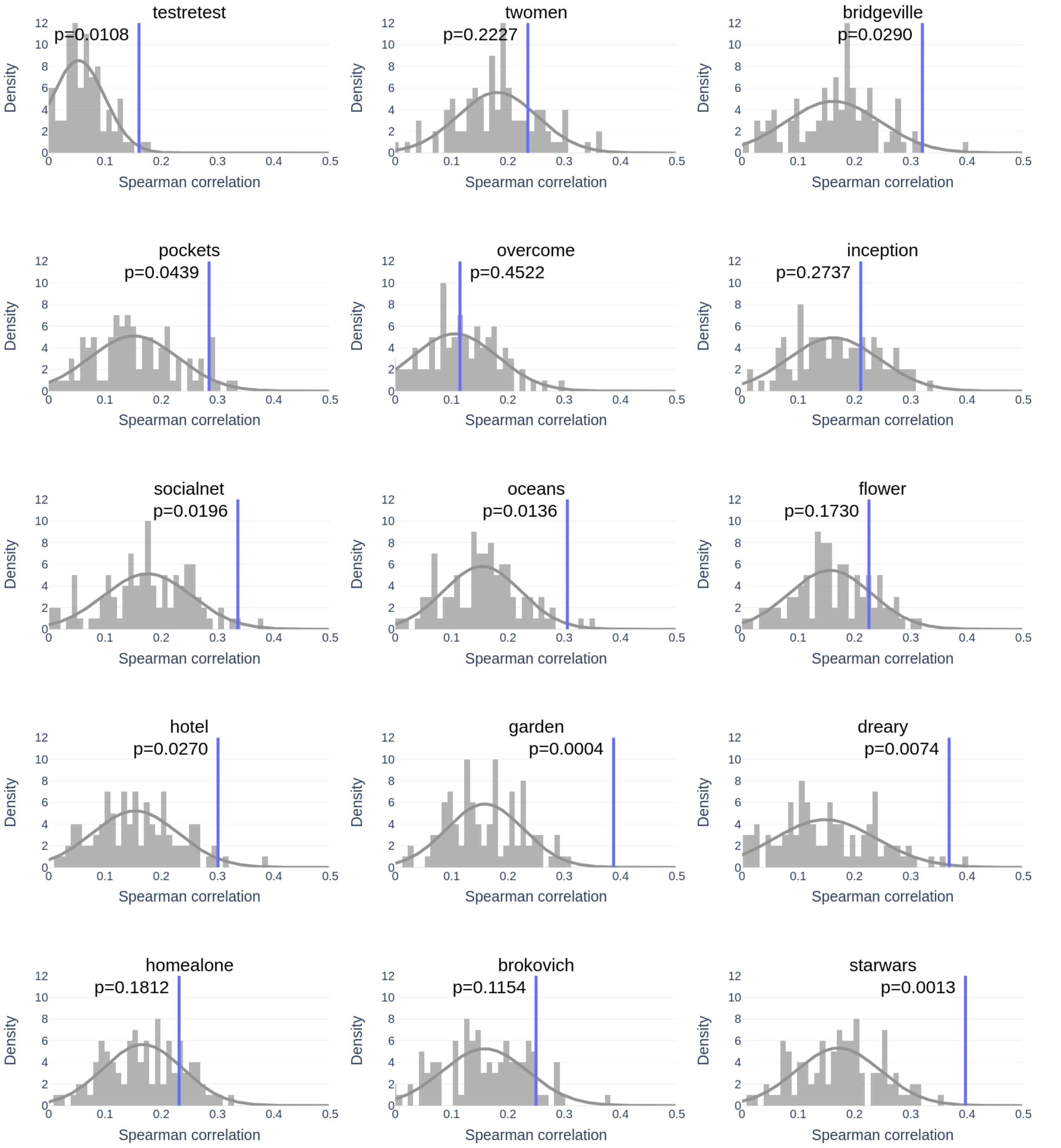

S3 Fig. Null distributions of fluid intelligence predictions. Vertical blue lines indicate the prediction based on actual data.
