# Supplementary material for: Learning brain dynamics for decoding and predicting individual differences
Source: PLoS Comput Biol. 2021 Sep 3;17(9):e1008943. doi: 10.1371/journal.pcbi.1008943 (PMC8445454; doi:10.1371/journal.pcbi.1008943)
Supplement: S4 Fig — Verbal IQ predictions for all movie clips. Conventions as in Fig 6 in the main text. (PDF) [file pcbi.1008943.s004.pdf]

S4 Fig. Verbal IQ predictions for all movie clips.

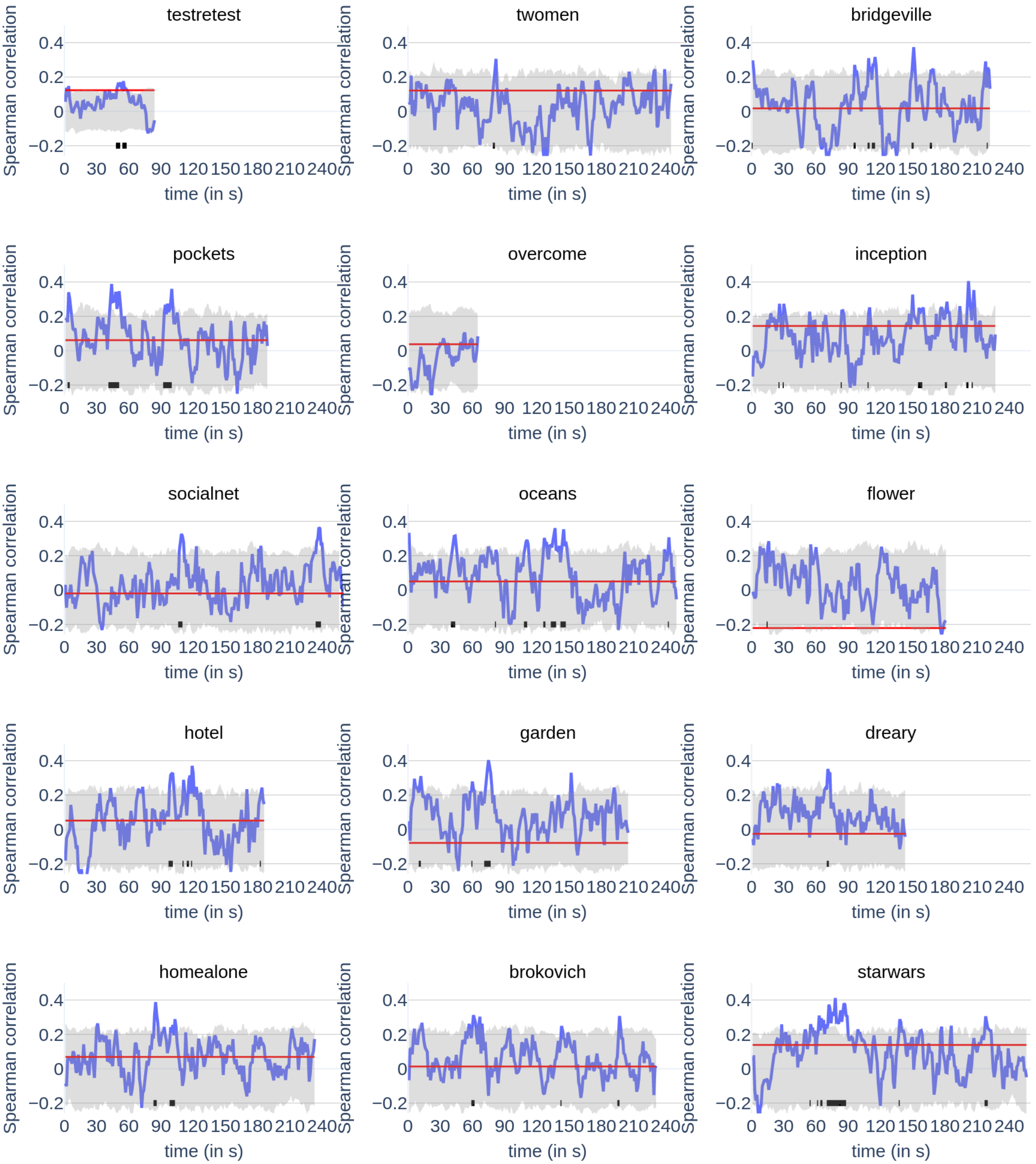

S4 Fig. Verbal IQ predictions for all movie clips. Conventions as in the Fig 6 in the main text.
