# Supplementary material for: Learning brain dynamics for decoding and predicting individual differences
Source: PLoS Comput Biol. 2021 Sep 3;17(9):e1008943. doi: 10.1371/journal.pcbi.1008943 (PMC8445454; doi:10.1371/journal.pcbi.1008943)
Supplement: S1 Appendix — (PDF) [file pcbi.1008943.s008.pdf]

S1 Appendix

Gated Recurrent Units We employed recurrent neural networks with Gated Recurrent Units (GRUs) which overcome challenges in learning temporal information i) by adaptively updating temporal history, and ii) by resetting/dropping temporal history that is irrelevant for future predictions [1, 2].

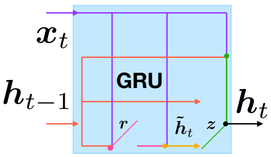

Fig. Formal specification of a GRU unit. The input, $\boldsymbol{x}_t$, and previous GRU output, $\boldsymbol{h}_{t-1}$, together generate a reset gate vector, $\boldsymbol{r}$, and an update gate vector, $\boldsymbol{z}$.

Given the input, $\boldsymbol{x}_t$, and previous output vector, $\boldsymbol{h}_{t-1}$, GRU units compute a reset gate vector, $\boldsymbol{r}$, and an update gate vector, $\boldsymbol{z}$:

$$\begin{aligned} \boldsymbol{r} &= \sigma\left(W_r \boldsymbol{x}_t + U_r \boldsymbol{h}_{t-1} + b_r\right), \\ \boldsymbol{z} &= \sigma\left(W_z \boldsymbol{x}_t + U_z \boldsymbol{h}_{t-1} + b_z\right), \end{aligned} \tag{1}$$

where W_r, U_r, W_z, U_z are weight matrices and b_r, b_z are bias vectors that are defined during the learning phase. The symbols $\sigma(\cdot)$, $\tanh(\cdot)$, and $\odot$ represent the sigmoid activation function, the hyperbolic tangent function, and the Hadamard product, respectively.

The two gate vectors are then used to compute a candidate state vector:

$$\tilde{h}_t = \tanh\left(W_h \boldsymbol{x}_t + U_h\left(\boldsymbol{r} \odot \boldsymbol{h}_{t-1}\right) + b_h\right), \tag{2}$$

using weight matrices W_h, U_h and bias vector b_h, which are defined during the learning phase ($N_{\tilde{h}} = 32$ dimensions). The reset vector, $\boldsymbol{r}$, controls the amount of information from the past output, $\boldsymbol{h}_{t-1}$, used to compute the candidate activation. Thus, when $\boldsymbol{r}$ was close to zero, the prior memory, $\boldsymbol{h}_{t-1}$, was deemphasized and $\tilde{\boldsymbol{h}}_t$ was "reset" with information from the current (brain signal) input, $\boldsymbol{x}_t$. Finally, the update gate vector, $\boldsymbol{z}$, governs the fraction of the previous state activation to be carried forward:

$$\boldsymbol{h}_t = (\mathbf{1} - \boldsymbol{z}) \odot \boldsymbol{h}_{t-1} + \boldsymbol{z} \odot \tilde{\boldsymbol{h}}_t. \tag{3}$$

Each GRU unit has its own reset and update gate units, thus allowing each unit to learn dependencies over multiple time scales. Units that learn long-term dependencies have their update gates frequently active, while those that learn shorter-term dependencies have their reset gates frequently active.

References

1. Cho K, van Merrienboer B, Gulcehre C, Bahdanau D, Bougares F, Schwenk H, et al. Learning Phrase Representations using RNN Encoder-Decoder for Statistical Machine Translation. arXiv:14061078 [cs, stat]. 2014;.

2. Gers FA, Schmidhuber JA, Cummins FA. Learning to Forget: Continual Prediction with LSTM. Neural Computation. 2000;12(10):2451–2471.
