# Supplementary material for: Learning brain dynamics for decoding and predicting individual differences
Source: PLoS Comput Biol. 2021 Sep 3;17(9):e1008943. doi: 10.1371/journal.pcbi.1008943 (PMC8445454; doi:10.1371/journal.pcbi.1008943)
Supplement: Attachment — Submitted filename: GRU_plos_responses_revision1.pdf [file pcbi.1008943.s009.pdf]

Learning brain dynamics for decoding and predicting individual differences

Joyneel Misra[1‡], Srinivas Govinda Surampudi[1‡], Manasij Venkatesh[1], Chirag Limbachia[2], Joseph Jaja[1], Luiz Pessoa[1,2*]

1 Department of Psychology and Maryland Neuroimaging Center, University of Maryland, College Park, MD, USA
2 Department of Electrical and Computer Engineering, University of Maryland, College Park, MD, USA

‡Authors with comparable contributions listed in random order

Please consider the revised version of our manuscript. Based on the revision work, we would like to seek permission to change the order of the authors, as indicated above.

We have now extensively revised our manuscript based on the excellent and constructive feedback from the reviewers (we know this is a cliché but we mean it). In addition to clarifying multiple issues, providing a more in-depth discussion of the results, we also performed an additional *lesion analysis* that complements the original saliency analysis. Combined, we believe the saliency plus lesion analyses illustrate how the model has the potential to advance understanding of spatiotemporal brain signals.

We note that classification accuracy values have now been slightly adjusted so that all of them are the average accuracy *after* the initial transient period of 90 seconds (see Figure 3). Previously, some of the values were inconsistently reported. We note that the changes are minor and do not alter any conclusions.

To facilitate the review, in the main text we indicate in the origin of change based on the number points below.

1 Reviewer 1

> Thank you for inviting me to review this manuscript by Pessoa and colleagues, in which the authors train a GRU with functional MRI data in order to classify different movie clips and task states, while also retaining the ability to track low-dimensional trajectories over time. Overall, I found the study to be of interest, however there were a number of sections that I believe would benefit from more explicit justification and expansion.

Major Comments

> **(1.1)** The authors may wish to provide some insights into why a GRU architecture was chosen over other potential options (e.g., vanilla feed-forward networks; convNets; LSTMs; etc). I appreciate that they used two of these as benchmark networks, however it would benefit the reader if the reasons for including them (or not) were made more explicit.

Our goal was to use a recurrent neural network based model, such as GRU- or LSTM-based architectures, to learn spatiotemporal information. These, and other,

architectures likely behave comparably, as observed across multiple applications [1]. We note that in some non-exhaustive experiments with LSTMs, we observed similar findings as reported in the manuscript. **Lines 126-130** now briefly discuss this point.

> **(1.2)** I apologise if I missed something, but how does training a GRU with an extra fully-connected hidden layer lead to low-dimensional representations of the data? Is it simply due to the fact that the added hidden layer has a smaller number of nodes? And if so, how did the authors determine precisely how low-dimensional to make this hidden layer?

Dimensionality reduction is obtained via two steps in our model. Because the dimensionality of the input ($N_x = 300$, in the movie dataset) is much higher than that of the hidden layer ($N_h = 32$), this accomplishes an initial amount of dimensionality reduction. This initial step is non-linear. However, we included a separate stage of linear dimensionality reduction that was obtained by adding an extra DRFC (fully connected) layer (see Figure 1B). The reason we did that was to have a modular architecture that could be probed as desired. For example, although it wasn't necessary, the dimensionality of the input layer could have been much larger to allow it to learn the dynamics at a high accuracy level, say $N_h = 100$. The additional DRFC would then allow one to investigate the extent to which this learned representation could be reduced in terms of dimensionality. We explain the above points in **lines 177-186**.

> **(1.3)** Fig 3. The results of Figure 3 are mildly surprising. Were the authors not surprised that their different test benchmarks performed so poorly, particularly when the only major differences between GRUs and ANNs/ConvNets is related to local nodal recurrence. It was also slightly strange that each of the null networks was strongly above chance (i.e., greater than 8%) but also hovered around the same value (40%). What is the significance of this value?

We did find it surprising that the feedforward network performed at a level comparable to that of temporal convolutional networks with windows of 5 (36.22%) and 10 data (41.56%) points (note that these values are slightly adjusted from those originally reported because of an inconsistency in reporting, as noted in the introduction to this letter). However, increasing the size of the window to 40 increased classification up to 54.85%. See the revised Figure 3.

As noted, the feedforward system performs well above chance (44.86%). This findings indicates that the classification problem can be solved without temporal information, consistent with the results of temporal shuffling (54.14%). However, the learning task was far from trivial, and our full model was unable to surpass 90% accuracy (recall, however, that our goal was not to seek maximum accuracy). As another example, consider that training a multinomial logistic regression model was unable to learn the task (8.10% accuracy). We revised Figure 3 and added **lines 377-380**.

> **(1.4)** Fig 4. Can the authors provide any intuition as to why the trajectories of the different movie clips arise from the origin of the 3D plot? The authors interest in fluid intelligence and fMRI may be augmented by reference to both https://doi.org/10.1038/nn.4135 and https://doi.org/10.1038/s41593-018-0312-0

Trajectories reflect temporal accuracy obtained by the GRU classifier (see Figure 3). Initially, accuracy is low and trajectories are very close to one another, hence the "origin"; as classification improves with time, discrimination among clips is higher and the corresponding trajectories more distinct as shown by the results in Figure 4B. We clarify this point in **lines 418-422**. We also now cite the suggested papers.

2 Reviewer 2

In this work, Pessoa et al. test a neural network architecture to decode fMRI-based brain dynamics and classify brain patterns elicited by movie-watching and a continuous experimental paradigm. They explore the associations between the employed GRU network and dimensionality reduction, behavior and brain activation through salience maps. The manuscript is generally clearly written and logically implemented. However, some of the characteristics of this methodology should be more clearly explained and motivated, before I can recommend this work for publication. Please find my comments below.

Major comments

(2.1) One of the goals of using this approach was to exploit the temporal richness of the fMRI brain signals. However, as the authors acknowledge, ”temporal shuffling of the time series still allowed the GRU classifier to attain classification at around 50% correct”. In other words, breaking temporal information still gives 50% accuracy after the GRU modeling procedure. Isn’t that worrisome? As a ”sanity” check, could the authors include a resting-state condition in the data pool, to see how it will affect the classification results?

This concern is similar to that of Reviewer 1, Major concern 3. Temporally shuffling the data removes temporal dependencies, causing performance to drop to 54.14%. This value is relatively close to that obtained with our feedforward classifier (44.86%; a feedforward architecture with 4 hidden layers attained 48.09% (not reported in the paper)). Thus, we believe that brain signals tied to *spatial* movie information likely support this level of performance. See the changes to section “Is temporal information necessary for clip prediction?”, and in particular **lines 388-390**.

(2.2) If I understood Fig. 4 correctly (I’d recoomend to improve the caption of 4C), the trajectories in 4A) are based on the first 3 PCA components of the GRU outputs h_t, which already allow for a high classification accuracy (60%). However, these very same 3 components have very little to do with the original brain signal (they only explain 2% of the variance of the original data!). This is somewhat in line with the common viewpoint of considering neural networks architectures as ”black box operators”, that output complicated non-linear projections of original signals, which are then harder to trace back. Therefore, I don’t understand how informative are the trajectories in 4A), since they have very little to do with the original brain signal, and are only related to the specific GRU architecture. This point should be better elaborated in the manuscript, to warn the reader on potential misleading interpretations of these NN classifiers.

These are very important points that certainly deserve further discussion; we thank the reviewer for bringing them up. In terms of the black box approach, we have added substantially to the Discussion (see **lines 562-574**). To further address the point, we performed a new lesion analysis, which was combined with the original saliency analysis. We believe our results illustrate how it is possible to map what the systems is learning to the signals and functions of sets of brain regions, this “opens the black box” to a good extent. Please see the new lesion analysis (Methods: **lines 247-253**; Results: Figure 6 and **lines 451-476**; Discussion: **lines 575-597**).

(2.3) On a related note, this also makes me wonder: how accurate is the approximation in equation 5), on which the brain salience maps were based?

The approximation in the original Equation 5 was provide as a way of motivating the idea of the saliency measure. We have now rewritten this part to make explicit the fact that the approach in fact does not involve an approximation. We apologize for the confusion. See the largely revised section "Saliency maps: Explaining model predictions via sensitivity analysis" starting at **line 211**.

(2.4) Similarly, what is the advantage of this complicated GRU-based model for the voxel prediction maps depicted in Fig. 7 with respect to, for instance, a simple GLM-based (SPM style) analysis? Wouldn't the GLM approach give you the same brain maps shown in Fig. 7?

We believe our approach is considerably different from the traditional GLM. Although the latter is flexible and one could use it in different ways, it typically refers to a *univariate* procedure that looks at the match of an entire fMRI time course to a predicted waveform, typically by convolving known condition regressors with the canonical hemodynamic filter. Our goal was to develop an approach to discover *multivariate* patterns that can learn specific temporal dependencies (given a target problem to be learned), so multivariate and temporal.

In the context of Fig. 8 (original Fig. 7), a GLM analysis would likely provide comparable information. But in this case, the paradigm is rather simple, as there are block-like periods of circle approach vs. circle retreat. The model we describe is clearly best suited to more complex designs. In fact, we developed the method after using GLMs for over 20 years exactly to try to analyze more complex designs we are studying now!

(2.5) I know that in our community there is the increasing need of connecting new computational models with behavior and/or clinical scores. However, I really have trouble understanding why the way a person watches Star Wars or Home Alone should be predictive of that person's "fluid intelligence". The current manuscript lacks a proper discussion of these results. I would appreciate a more in-depth reasoning on these "neuroscientific" findings, rooted in the state-of the art of naturalistic task analysis. In other words, it is clear that the GRU black box is very effective at classifying different naturalistic tasks, but what do we learn about the brain that we did not know before?

We strongly agree that further discussion of the results is important. Based on the new combined saliency and lesion analysis, we have expanded on the neuroscientific findings. See section "Saliency and lesion analysis" in the Discussion (**lines 606-622**).

Minor comments

(2.6) Was band pass filtering applied to the fMRI data? What about Global signal regression? Would those impact the classification results?

We did not apply band-pass (BP) filtering or perform global signal removal (GSR). We tested the impact of those steps on our results but the impact was negligible:

	**Original accuracy**	**BP filtering**	**GSR**
FF	44.86%	44.86%	45.08%
TCN 5	36.22%	36.22%	36.30%
TCN 10	41.56%	41.56%	42.23%
GRU	89.46%	89.26%	89.32%

(2.7) Moving-circles data: how were the voxels selected?

We analyzed data at the voxel level from the right anterior insula, which corresponds to region numbers 230 and 231 of the `Schaefer-300` 17-network parcellation (**lines 97-99**).

(2.8) Behavior. "Because of multiple comparisons across time, we performed an additional test considering all time points simultaneously." It is not clear how the authors correct for multiple comparisons. Please clarify.

Our original text wasn't sufficiently clear, and failed to make it clear that multiple comparisons was addressed because the statistic proposed performs a *single* test that evaluates the following: is there a temporal window of length l that exhibits as high an average correlation under the null scenario? The null scenario in question is one in which participants are shuffled (i.e., the fMRI and fluid intelligent were randomly associated, not based on participant ID). As one test is performed, correction is not necessary. We clarified the text as outlined in lines **312-320**; see also the green line added to the current Figure 7.

3 Reviewer 3

The paper presents a spatiotemporal analysis framework for dynamic classification, regression and visualization of fMRI data. The authors develop a deep learning architecture based on GRUs (a recurrent neural net architecture) using three related components: a classifier to predict the class of stimuli, an encoder for dimensionality reduction to perform visualization and a decoder. They also present saliency maps which enable an interpretability of the model and provide information on the importance of different brain regions / voxels to the classification. This is an advantage of the approach compared to other nonlinear techniques which cannot map back to the original input space. I enjoyed reading the paper and believe it is an important contribution of interest to the readers of PLOS Computational Biology.

Major comments

(3.1) The authors should comment in the paper why they choose to train three separate networks instead of training an end-to-end neural network for classification, encoding and decoding, i.e. an alternative architecture could have been a jointly trained autoencoder and classifier but perhaps this would be less stable to train or result in poorer performance. In addition regarding the encoder, since it shares the GRU with the classifier (these aren't actually fully separate), this should be made more explicit in the figure.

The main motivation was to develop a model with considerable *modularity*, such that specific components of the architecture could be exchanged based on research preferences and, critically, based on future neural network developments. See **lines 177-186**. Furthermore, we have added text to clarify that the GRU part is shared with the classifier (Figure 1).

(3.2) Related work: the introduction should also briefly discuss how this paper relates to recent work on dynamical functional connectivity.

We added the following text (**lines 22-25**): "Another strategy to make use of temporal information capitalizes on dynamic functional connectivity, and how it is related to different conditions and individual differences [17-20]."

> **(3.3)** The authors make the point that decoding is typically static, based on for example reducing the activity to a functional connectivity matrix or averaged activity. A static method should be added as a benchmark to the classification task, similar to including CPM as a static baseline for predicting fluid intelligence and verbal IQ.

We note that the feedforward method included originally is a static method, as classification is performed at every time point, separately. In addition, we now also include another static method, namely multinomial logistic regression, which was unable to learn the classification problem (8.1% correct). This method is now mentioned in **lines 369-370**; see also Figure 3.

> **(3.4)** For the TCN classifier, increasing the length of the kernel from 5 to 10 time steps improved performance. Does a further increase to larger kernel widths (20?40?) further improve classification accuracy? This also relates to the author's discussion on spatiotemporal representations that long-term dependencies are essential.

Classification accuracy with temporal convolutional networks improved with window size, but at a substantial cost in terms of number of parameters. For example, with a window width of size 40, accuracy reached almost 55% but used essentially 10 times as many parameters as the GRU. We now note the result with sizes 10 and 40 in **lines 377-380, and lines 388-390**, and added the latter to Figure 3. The table below shows results when varying kernel width:

**Kernel width**	**Accuracy**	**Number of model parameters**
5	36.22%	37915 ($\approx$ 32559 GRU parameters)
10	41.56%	75415
20	47.08%	150415
30	50.74%	225415
40	54.85%	300415

> **(3.5)** Regarding the low-dimensional trajectories as well as trajectory distances, what can be learned from this visualization, i.e. what is the aim of visualization? Is the organization of video clips meaningful in any way?? The result that classifying on the low dimensional space (3-10 dimensions) yields classification accuracy close to the full dimension is interesting as it indicates the intrinsic dimensionality of the data.

As the task learned by the network was to simply classify/separate the clips, we don't believe the 3-D trajectories capture information in a deeper way, except to reflect the fact that classification is poor initially and increases rapidly during the first 60 seconds and less rapidly up to 90 seconds or so. In other words, even in 3-D they effectively separate. We believe our expanded discussion in the context of question 2 by Rev 2 also addresses the present question when we discuss "representational content" in particular (see **lines 562-574**).

Minor comments

> **(3.6)** Line 12 "to infer mental processes and processes from brain signals." Missing word?

We rephrased the text in **lines 11-12**.

> **(3.7)** GRU – main text should mention detailed explanation as in supplementary

We would prefer to keep the GRU description in the supplementary material as the Methods section is quite long already. However, we are happy to bring it into the main text if the reviewer and editor find it appropriate.

> **(3.8)** Line 131 N_y should be N_h

We have updated **line 151**.

> **(3.8)** Line 148 – should be layers by units

We have updated **line 168**.

> **(3.10)** Line 256 – how was the threshold of 0.2 chosen?

The threshold was set to 0.2 as many analyses of functional connectivity use this threshold, but the value was arbitrary. Because we don't investigate this issue further, we have rephrased our results to indicate that they are *comparable* to (not better than) those of CPM.

> **(3.11)** Table 1 – add number of layers

We have updated Table 1.

> **(3.12)** Line 284 - "were obtained with not used" – missing word?

We have updated **line 348**.

> **(3.13)** Red line missing in Fig S4 for flower clip

We have updated Fig S4.

4 Reviewer 4

> The authors propose using modern recurrent neural networks (in this case, a network with gated recurrent units; GRUs) to analyze spatial-temporal brain data in rich, continuous experimental settings like movie watching. Considering most fMRI work is based on static images, this seems like a potentially interesting approach to reveal what previous work could not.

> Overall, the authors did a lot of work, but did not sufficiently motivate and explain the methods in a way that convinced me that we learnt much new - or what the new part was. The authors need to explain and motivate their approach, what their findings are, and why this is the way to go (at least for this kind of data). Below, I note major and minor points that I hope will be helpful to improve the manuscript.

Major comments

> **(4.1)** General point on motivation and introducing the method. It was not clear to me what we benefit from this analysis from a broad perspective, or from the specific analysis steps. There is a brief general note on what they will do and why (e.g. decode, reduce dimensionality), but not clear what the conceptual significance is (test what kind of information there is some brain area, explain what temporal information is capture and why it is interesting).

We have expanded about the motivation of our approach in the Introduction (see **lines 56-68**). In addition, we have considerably expanded the Discussion section, which we hope motivates our approach in greater depth.

> **(4.2)** Interpretation problem from nonlinear decoders. What can we say from a non-linear decoder? Standard MVPA is linear - and the point is if you can read out information from a certain brain area from a linear decoder, the content is there in an explicit way. For example, you can decode anything from the retina if you use a non-linear decoder, but that doesn't mean everything is represented there. For example, see Tong and Pratte 2012, p487, DiCarlo et al., 2012 on tangled representations, Kriegeskorte & Kreiman 2011.

We agree that nonlinear decoders have to be used carefully. In fact, our goal was to "decode" only insofar as it would provide potentially meaningful information about the underlying brain processes. Thus, our original saliency analysis sought to identify important sets of regions. The new lesion analysis, as well as its combination with saliency information, sought to develop this point further. In a more general setting, we believe these types of findings provide potential hypotheses that can fruitfully lead to more targeted experiments. We have substantially altered the manuscript accordingly: **lines 62-68, 247-253, 451-476, 562-597**, and Figure 6.

> **(4.3)** What does the decoder tell us? What did we learn? The classifying decode which movie clip it is across participants. This tells us the brain holds some info about movie clip. Is this the conclusion? What have we learnt? Is it based on visual, auditory information, or semantic content? More generally: what would decoding of a movie clip mean - we probably don't care that area X represents encodes movies, or distinguishes different movies... What is the important point? Although the authors argue that the method give spatial and temporal insights, there doesn't seem to be any spatial or temporal interpretation since it uses all the data to construct the classifier. Training on temporal data then testing on specific time points - does this give any info on that specific time point, is there a problem with the classifier having inputs/info from other timepoint? The authors use saliency maps for finding interesting spatial or temporal points, but it's unclear if this is meaningful (see point below on saliency maps).

A central aim of the paper was to describe the method, and from the reviews we see that we didn't motivate it sufficiently. The original saliency analysis is now combined with a lesion analysis (**lines 451-476**) to illustrate the kinds of knowledge we can gain from the approach. See also the revised section in the Discussion (**lines 562-597**). About the idea of training on part of the time series and testing on other temporal segments, we agree that this is an interesting direction. In fact, we plan on applying the approach to predicting future behavior/states, so we prefer to leave those for a follow-up study to keep the present paper more manageable in terms of scope/size.

> **(4.4)** Why GRUs, why is it good for the current setting? Lack of a motivation/explanation. Typically, GRUs are used to predict the next time step. Here it seems like it tries to do that too, but then prediction is then on a 15-way classifier to classifier movie clips across participants. Not clear how the temporal prediction, applied to across subjects analysis and not predicting time, helps. Authors need to clarify this - and maybe it is an important method point that I missed.

The goal was to employ a recurrent architecture that could effectively learn the long-term history of the input. Although it is common for applications of GRU/LSTM systems to use the last point as the bases of prediction, as our central goal was not prediction, we were interested in characterizing performance as a function of time. In fact, that was related to our objective of studying saliency as a function of time, with the more genaral objective of determining certain temporal segments that could be informative. For example, for some regions in Fig. 5, there's a second "bump" around 35-50 seconds that provides additional classification information. In addition, for the left inferior frontal cortex, although the large peak is transient for Starwars, the entire window until around 50 seconds is informative (better than chance). The saliency analysis can also be coupled with a "silencing" analysis during particular time periods, although we did not include that type of analysis in the current version to keep the focus of the paper.

> **(4.5)** Saliency maps - the paper authors cite was for images in convnets, a visualizing technique based on images which usually finds where the 'main object' is. Is this transferrable to brain data? What is a 'salient' activation/timepoint in the brain in this case? Input feature is time and/or space - what would a salient timepoint mean? Strong activation, or strong decoder? Is it just a (statistical) way to extract the strong areas/timepoints or something more? Is it related to saliency in the clips (probably not?)?Not much details in the results and discussion. Would be good to have this from the methods and to fully motivate the meaning and purpose of this measure.

Our original description of saliency wasn't sufficiently clear. Our saliency method is part of a broader approach called "sensitivity analysis", which is regularly used applications of machine learning models [2–5]. Saliency maps represent spatio-temporal patterns of the input that the decoder highlights as important for the problem at hand (such as 15-way movie classification). Combined with the lesion analysis, the approach has the potential to uncover useful information about the brain. We hope that the substantially revised paper makes a good case for this.

> **(4.6)** Dimensionality reduction - this is non-linear, so again, this seems like there might be a problem for interpretation, especially when it is used to test if you can decode from the low-dimensional space - i.e. same problem as a non-linear decoder above. Furthermore, this is now a non-linear space - which could be high dimensional in linear space? Separately - dimensions increased variance explained - is it still a low-d manifold? Looks like it keeps increasing. That the first few dimensions take much of the variance is common (e.g. in PCA), and here it's non-linear which might mean it's more. This is not so much an issue of method but interpretation. Maybe more important: it is 10d with time - is this not much higher dimension than 10?

The point raised by the reviewer ("10d with time") is insightful and deserves to be highlighted, and we do so explicitly in **lines 553-558**.

(4.7) Predicting 'behavior' (e.g. IQ scores) - why is this done over time? Is it actually predicting a single score over time? Why would you predict something multiple times when there is only 1 score (stats point) - it seems more valid (and powerful) to predict one score per participants with all the data. More importantly, why would you theoretically predict that it varies, and correlates with movie watching?

As the formalism developed is temporal, we thought it would be valuable to look at prediction temporally, as discussed in **lines 615-622**. In addition, we have developed an additional test, which we call "oracle test", that performs a single prediction based on guessing the best time from the training set and applying it to the test set (**lines 328-338**).

Minor comments

(4.8) Discussion is very short and basic, reiterating the results without much interpretation of what they mean. If the contribution is more about a method, more discussion should go into the methods and comparison with previous methods. If it's about prediction (in the ML sense), then they can also be more clear about that, without interpretation.

Based on the reviewers' feedback, we have considerably expanded the Introduction and Discussion text. Hopefully, this fills in some of what the reviewer has in mind.

(4.9) The GRU model does better than other models that don't take into account time. These are some good comparisons. However, it was unclear to me if the GRU was therefore more complex (it is in the sense that it takes into account temporal info, so maybe that's unavoidable) so did better, and if there are more parameters (which would be unfair).

In terms of parameters, GRU is not more complex, as indicated in the original text (see **lines 377-380**).

(4.10) "As our goal was not necessarily maximizing model performance, we explored a small set of potential architectures by varying the number of hidden layers and number of units per layer "If it's not, what's the purpose of testing multiple models? Having multiple models show similar effects could be informative to show that it's reliable across different model settings, but it seems like the authors did choose the best model. Why in the HCP 1 layer enough and in the moving circles task 3 layers needed? Classification task was harder, probably less information (e.g. visual). But this is now more difficult to interpret. What does this tell us - does it tell us anything apart from decoding is harder because there less information? Then making the model more complex you can decode more - this is obvious, but not clear how it's interpretable neurally.

We meant that we did not seek to optimize the model exhaustively. We did however try to obtain a setting that was "good enough". The moving circles was clearly much more challenging for the GRU to learn, likely because only voxels from one part of the insula were used.

(4.11) Train on subset of participants, test on others (100 train, 76 test). This is fine but why these numbers? Worth justifying in the text (e.g. more training data can be more desirable than split half).

The rationale was to have a little more data for training, as indicated by the reviewer. We added an explanation in **lines 110-111**.

> **(4.12)** Threat vs safe - it seems like the brain region would have come up in a more traditional univariate and/or multivariate (e.g. linear SVM) analysis as well. What is added here?

This question is similar to that asked by Reviewer 2; please see our reply to 2.4 above. The central point is that we sought to devise a temporal technique to investigate spatiotemporal brain signals. Admittedly, the moving circles paradigm is somewhat simple ("block like"), and the model is better suited to more complex designs.

References

1. Chung J, Gulcehre C, Cho K, Bengio Y. Empirical Evaluation of Gated Recurrent Neural Networks on Sequence Modeling. arXiv:14123555 [cs]. 2014;.

2. Gevrey M, Dimopoulos I, Lek S. Review and comparison of methods to study the contribution of variables in artificial neural network models. Ecological Modelling. 2003;160(3):249–264. doi:10.1016/S0304-3800(02)00257-0.

3. Baehrens D, Schroeter T, Harmeling S, Kawanabe M, Hansen K, Müller KR. How to Explain Individual Classification Decisions. The Journal of Machine Learning Research. 2010;11:1803–1831.

4. Lanchantin J, Singh R, Wang B, Qi Y. Deep motif dashboard: visualizing and understanding genomic sequences using deep neural networks. In: Biocomputing 2017. WORLD SCIENTIFIC; 2016. p. 254–265. Available from: `https://www.worldscientific.com/doi/abs/10.1142/9789813207813_0025`.

5. Khan J, Wei JS, Ringnér M, Saal LH, Ladanyi M, Westermann F, et al. Classification and diagnostic prediction of cancers using gene expression profiling and artificial neural networks. Nature Medicine. 2001;7(6):673–679. doi:10.1038/89044.
